# Gender perspective of dual diagnosis

**DOI:** 10.1192/j.eurpsy.2025.122

**Published:** 2025-08-26

**Authors:** J. Tirado Muñoz

**Affiliations:** Department of Psychology, Universidad Europea de Madrid, Madrid, Spain

## Abstract

**Abstract:**

Dual diagnosis, the co-occurrence of substance use disorders (SUD) and mental health conditions, presents unique challenges influenced by gender-related factors. This narrative review explores the role of gender in the epidemiology, treatment access, environmental factors such as intimate partner violence, clinical features, and outcomes of dual diagnosis, highlighting structural and social determinants that exacerbate disparities. Women and gender minorities with dual diagnosis face heightened stigma, higher rates of trauma, and significant barriers to healthcare, often resulting in delayed treatment and poorer prognoses. Additionally, traditional gender roles and caregiving responsibilities influence coping mechanisms and treatment adherence. Despite increasing awareness, research on gender-responsive interventions remains limited. This review underscores the need for gender-sensitive approaches in dual diagnosis treatment, advocating for integrated, trauma-informed care that addresses the specific needs of diverse gender identities.

**Disclosure of Interest:**

None Declared

